# Trichostatin A, a Histone Deacetylase Inhibitor, Alleviates Eosinophilic Meningitis Induced by *Angiostrongylus cantonensis* Infection in Mice

**DOI:** 10.3389/fmicb.2019.02280

**Published:** 2019-10-04

**Authors:** Yanhua Zhang, Hui Xie, Wenyan Tang, Xingda Zeng, Yu Lin, Lian Xu, Lihua Xiao, Jun Xu, Zhongdao Wu, Dongjuan Yuan

**Affiliations:** ^1^Department of Parasitology, Zhongshan School of Medicine, Sun Yat-sen University, Guangzhou, China; ^2^Key Laboratory for Tropical Diseases Control (SYSU), Ministry of Education, Guangzhou, China; ^3^Provincial Engineering Technology Research Center for Diseases-Vectors Control, Guangzhou, China; ^4^Department of Pediatrics, The First Affiliated Hospital, Sun Yat-sen University, Guangzhou, China; ^5^College of Veterinary Medicine, South China Agricultural University, Guangzhou, China; ^6^School of Pharmaceutical Sciences, Sun Yat-sen University, Guangzhou, China

**Keywords:** trichostatin A, *Angiostrongylus cantonensis*, eosinophilic meningitis, inflammation, cognitive impairments, HDAC, NF-κB

## Abstract

Histone deacetylase inhibitor (HDACi) has been used in the treatment of neurodegenerative or autoimmune diseases. Angiostrongyliasis cantonensis caused by *Angiostrongylus cantonensis* infection is an emerging zoonosis of human eosinophilic meningitis or meningoencephalitis. Progressive neuronal apoptosis is the pathological basis of behavioral dysfunctions in angiostrongyliasis cantonensis. Neurological defects after anthelmintic treatment for angiostrongyliasis cantonensis are still common. In this study, we examined the effects of trichostatin A (TSA), a HDACi, on eosinophilic meningitis induced by *A. cantonensis* in mice. Intragastric administration of TSA significantly ameliorated brain injury and decreased cognitive impairments in mice at 15 days post-infection. TSA administration effectively reduced the inflammatory factor levels of iNOS, TNF-α, IL-5, IL-6, and IL-13 in infected mice. TSA treatment counteracted apoptosis with reduced expression levels of cleaved caspase-3, -4, -6, and RIP3 in *A. cantonensis* infected mice. In addition, TSA administration reduced total HDAC activity and increased the acetylation of histone H3 and H4 in the brain tissue of infected mice. The underlying mechanism of TSA on eosinophilic meningitis might be associated with decreased NF-κB p65 nuclear accumulation by inhibiting IκB phosphorylation. Furthermore, a co-expressive network of NF-κB p65 with 22 other genes was constructed according to our previous transcriptomic data in infected mice. We identified the correlations in the gene expression of *NF-*κ*B p65* with *Lrp10*, *Il12rb1*, *Nfkbia*, *Ube2n*, and *Ube2d1* in infected mice after TSA administration. Thus, TSA has a protective effect on the progression of eosinophilic meningitis induced by *A. cantonensis* in mice.

## Introduction

*Angiostrongylus cantonensis* (*A. cantonensis*), a rat lung nematode, is an infectious food-borne zoonotic parasite that can cause severe disease. Humans become infected with *A. cantonensis* by eating raw or improperly cooked freshwater snails containing the infectious third-stage (L3) larvae, and infection caused human eosinophilic meningitis or meningoencephalitis (Noda et al., 1987; Duffy et al., 2004; Tsai et al., 2004; Er-Hu et al., 2008; Mason, 2010). Human angiostrongyliasis cantonensis is endemic in South-east Asia, the Pacific Islands and the Caribbean (Lv et al., 2011). In the past 10 years, hundreds of cases and several outbreaks of this disease have been reported in endemic regions, especially in China (nine outbreaks in mainland China and three in Taiwan, China). The main pathological characteristic induced by *A. cantonensis* is eosinophilic meningitisis hemorrhage, vascular dilatation, focal necrosis with neuronal loss, and infiltration of inflammatory cells in brain parenchyma (OuYang et al., 2012). Our previous study showed that neuronal apoptosis might be the pathological basis of behavioral dysfunctions in rodents with *A. cantonensis* infection (Luo et al., 2017). In patients with angiostrongyliasis, neurological defects with persistent headache, paresthesia or hyperesthesia, nuchal rigidity, seizure, cognitive dysfunction, ataxic gait, and even unconsciousness after anthelmintic treatment are still common (Hidelaratchi et al., 2005). Thus, effective treatment of angiostrongyliasis cantonensis should include the exploration of additional agents for reducing neurological defects.

A key post-translational modification for regulating gene transcription is the acetylation of histones or other proteins (Johnstone, 2002; Yang and Seto, 2008; Giavini and Menegola, 2014). The level of protein acetylation is regulated by the activities of histone deacetylase (HDAC) and histone acetyltransferase (also named K(lysine) acetyltransferase, KAT). Recently, HDAC inhibitors have been reported to modulate the activity of nuclear factor-kappa B (NF-κB) in different disease models (Leus et al., 2016; Zhang et al., 2018). NF-κB is a central mediator of the immune and inflammatory responses and is involved in the transcriptional regulation of apoptosis-related genes (Greten et al., 2004; Place et al., 2005). HDAC inhibitor (HDACi) exhibits neuroprotective effects by reducing the expression of proinflammatory molecules such as p53 and NF-κB to mitigate neuronal apoptosis (Moreira et al., 2003; Kim et al., 2007; Shein et al., 2009; Leus et al., 2016). HDACi has a long history of usage in psychiatry and neurology as a mood stabilizer and anti-epileptics agent, and it is being studied as a mitigator or treatment for neurodegenerative diseases (Hahnen et al., 2008). In addition, HDACi decreases lipopolysaccharide (LPS)-induced inflammatory response by reducing inflammatory cell recruitment (Brogdon et al., 2007) and decreasing cytokine expression (Suh et al., 2010).

HDACs can be classified into three sub-classes named class I (HDACs 1, 2, 3, and 8), class II (HDACs 4, 5, 6, 7, 9, and 10), and class IV (HDAC 11) (Khan et al., 2008). Trichostatin A (TSA), a well-known HDACi, efficiently inhibits the deacetylation of class I, II, and IV HDACs to enhance histone acetylation and regulate the expression of cytokines (Menegola et al., 2006; Marks, 2010). TSA administration has neuroprotective effects on female neonatal mice following LPS/heat-inactivated (HI) treatment and correlates with improved long-term learning (Fleiss et al., 2012). TSA also has been considered a potential therapeutic agent against hepatic fibrosis and asthma (Van Beneden et al., 2013; Toki et al., 2016).

In this study, we evaluated the effects of TSA on the eosinophilic meningitis induced by *A. cantonensis* in mice. We also sought to explore the effects of TSA treatment on brain injury in mice.

## Materials and Methods

### Ethics Statement

All procedures involving animals conformed to the Chinese National Institute of Health Guide for the Care and Use of Laboratory Animals, and the protocol was approved by the Sun Yat-sen University Committee for Animal Research (No. 2016-104).

### Experimental Animals and Treatments

Male BALB/c mice (specific pathogen free, SPF) aged 6 weeks were supplied by the Center of Animal Experiments of Sun Yat-sen University. L3 larvae of *A. cantonensis* were collected to infect mice by intragastric administration as previously described (Xie et al., 2017). The experimental mice were randomly divided into six groups: non-infected group (control group), infected group, and infected mice that received treatments (four groups treated with 2, 5, 10, and 20 mg/kg TSA). Each group has 10 mice. Each mouse was infected with 50 L3 larvae except for the control group. The mice in the TSA treatment groups were intravenously injected with TSA (10% DMSO, Sigma-Aldrich, United Kingdom) at 1th day post-infection (dpi), and the control and infected group were treated with the same amount of DMSO as control. The mice were euthanized for further experiments at 15 dpi.

### Behavioral Testing With the Morris Water Maze

The Morris water maze test is currently the most frequently used method to evaluate learning and memory skills in mice following the protocol previously described (Vorhees and Williams, 2006; Crawley, 2007). The place navigation test was performed on 4 consecutive days, and each day, the mice were trained to find the platform in four quadrants. Animals that failed to find the platform within 60 s were gently guided to the platform to rest for 30 s. The spatial probe task, used to evaluate mouse memory retention, was given after 24 h following the last acquisition trial. The platform was removed from the pool, and the probe task was performed in the third quadrant. The escape latency, swimming tracks, velocity, percentage of time in the target quadrant, and number of times the mice crossed the annulus where the platform located were recorded by a video tracking system (XinRuan Tech, Shanghai, China).

### Histological Examination

The brains were harvested and immediately fixed in 4% paraformaldehyde and then processed according to standard procedures. Five-mm-thick paraffin-embedded brain sections were subsequently prepared and stained with hematoxylin and eosin (H&E). All tissue slices were examined with an Automatic Digital Slide Scanning system (AxioScan.Z1; Germany) and the pathological changes were evaluated with ZEN software (Zeiss, Jena, Germany).

### mRNA Analysis

mRNA expression levels were quantified using quantitative real-time polymerase chain reaction (qRT-PCR). Total RNA was extracted from brain tissue using TRIzol (Invitrogen) and reverse-transcribed to cDNA using a PrimeScript^TM^ RT Master Mix (TaKaRa, Japan) according to the manufacturer’s protocols. Gene expression was quantified with SYBR^®^ Premix Ex Taq^TM^ (TaKaRa, Japan) using the LightCycler^®^ 480 instrument (Roche Diagnostics, Switzerland). The primers used for RT-PCR are listed in Supplementary Table S1. PCR was performed with a reaction mixture with a total volume of 20 μL comprising the following: 10 μL of SYBR^®^ Premix Ex Taq^TM^ (2×), 1 μL of forward primer (10 μmol/L), 1 μL of reverse primer (10 μmol/L), 1 μL of template, and 7 μL of ddH_2_O. The reaction comprised the following steps: an initial denaturation at 95°C for 30 s, followed by amplification for 35 cycles at 95°C for 5 s and 60°C for 20 s. The relative mRNA expression levels of the target genes were normalized to those of the indicated housekeeping gene (β-actin) and were quantified using the comparative Ct method and the formula 2^–ΔΔCt^.

### HDAC Activity Assay

The HDAC activity was measured using an HDAC activity assay kit (GENMED, United States) according to the manufacturer’s instructions. In brief, 10 μL freshly harvested serum was incubated with HDAC assay substrate at 37°C for 1 h. After five washes, reagent was added, the reaction was maintained at room temperature for 15 min, and then the absorbance value was measured at 450 nm. A standard curve was performed according to the manufacturer’s protocol.

### NF-κB P65 Protein Nuclear Accumulation

Nuclear protein was isolated from brain tissues using the Minute^TM^ Cytoplasmic and Nuclear Extraction Kit (Invent Biotechnologies, Beijing, China) according to the manufacturer’s protocols. A total of 100 μL of nuclear protein extracts was used to determine the nuclear level of NF-κB p65 protein (Mouse NF-κB p65 ELISA kit, Cusabio, Wuhan, China) according to the manufacturer’s protocols. The results are expressed as pg/mL protein.

### Western Blotting

Protein expression in brain tissues was detected by western blotting. Fifty milligram protein was extracted from 100 mg brain tissue samples and subjected to sodium dodecyl sulfate-polyacrylamide gel electrophoresis (SDS-PAGE) according to standard methods. Then, proteins were electrophoretically transferred to a polyvinylidene difluoride (PVDF) membrane (Millipore, Germany) and blocked using 5% skim milk. The membranes were incubated overnight at 4°C with the following antibodies: anti-cleaved caspase-3, anti-IkBα (Proteintech, Wuhan, China), anti-phosphor-IkBα (Abscitech, Shanghai, China), anti-acetyl-histone H3, anti-acetyl histone H4, anti-NF-κB p65 (Cell Signaling Technology, Danvers, United States), and anti-GAPDH (Cell Signaling Technology, Danvers, United States) as the control, and then incubated with a secondary antibody for 2 h at room temperature. The membranes were visualized with an enhanced chemiluminescence (ECL) western blotting detection system (Amersham, United States). The changes in the protein levels were calculated using ImageJ software (Patel et al., 2011).

### Functional Interaction Network Analysis of NF-κB p65

Pearson correlation coefficients for NF-κB p65 with all other genes in our previous RNA-seq were calculated (Yu et al., 2015). Genes that were highly correlated with NF-κB p65 (absolute Pearson correlation coefficients cutoff: 0.85) were identified and submitted to the STRING database^1^ to retrieve potential interactors (medium confidence cutoff: 0.4) (Szklarczyk et al., 2015). Potential interactors were further analyzed and visualized using Cytoscape (v3.6.1) (Shannon et al., 2003). Then, a sub-network of genes interacting with NF-κB p65 was constructed. The JASPAR database^2^ was used to identify transcription factor binding sites (TFBS) in the promoter region of genes (Ho Sui et al., 2005).

### Statistical Analysis

All data are expressed as the mean ± SEM. One-way ANOVA was used to analyze the significance of the differences between groups, and *P* < 0.05 was considered statistically significant.

## Results

### TSA Treatment Ameliorated the Histological Changes in the Mouse Brain

**FIGURE 1:**
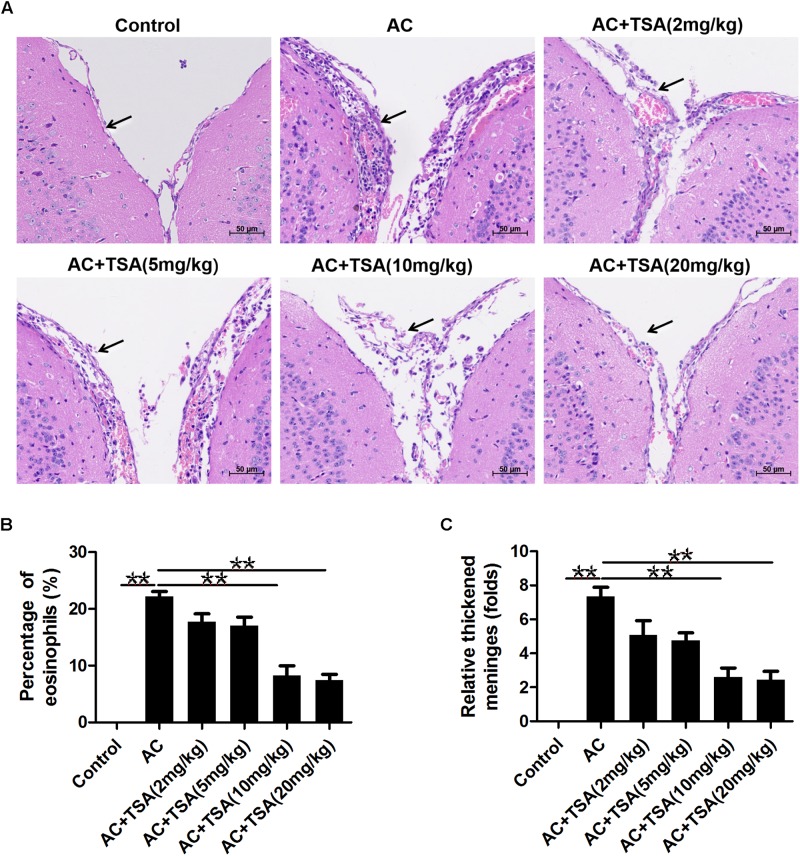
TSA treatments ameliorated brain meningitis induced by *A. cantonensis* in mice. **(A)** Pathological changes of mouse brain tissues in infected mice with TSA treatments. The arrow shows the meninges and infiltration of eosinophil cells. AC: *A. cantonensis* infection; AC + TSA (2 mg/kg), AC + TSA (5 mg/kg), AC + TSA (10 mg/kg), AC + TSA (20 mg/kg): *A. cantonensis* infected mice that received 2, 5, 10, and 20 mg/kg TSA treatment, respectively. **(B)** Percentage of eosinophil cells in the total cells of mouse brain tissue slices in each group. **(C)** Relative thickened meninges in mouse brain tissue slices in each group. The data are presented as the mean ± SD, *n* = 7. ^∗∗^*P* < 0.01.

We assessed the impact of TSA on eosinophilic meningitis induced by *A. cantonensis* in mice. Infected mice exhibited traumatic lesions and subarachnoid hemorrhage in the brain. HE staining revealed the meningitis with thickened meninges, infiltration of eosinophil cells, and inflammatory damages compared to the control (Figure 1). TSA treatment significantly ameliorated the eosinophilic meningitis in mice infected with *A. cantonensis* in the 10 and 20 mg/kg TSA treatments groups; however, this effect was not obvious in the 2 and 5 mg/kg TSA treatments groups (Figure 1). Thus, there was a dose-dependent effect of TSA treatments on mouse eosinophilic meningitis from *A. cantonensis* infection, and based on these data, the 10 and 20 mg/kg TSA dose were chosen for all further studies.

### TSA Treatment Decreased Cognitive Impairments in Mice Caused by *A. cantonensis*

**FIGURE 2:**
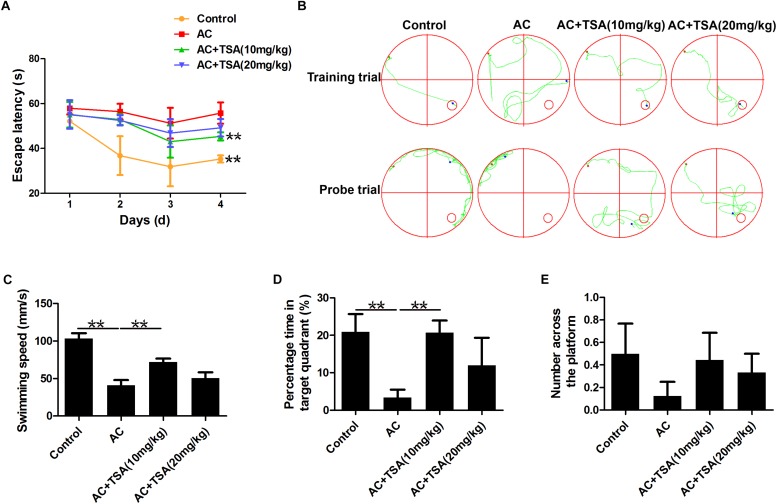
TSA treatment decreased cognitive impairments caused by *A. cantonensis* in mice. **(A)** Escape latency of each group in four trains (^∗∗^*P* < 0.01, control versus AC, AC + TSA (10 mg/kg) versus AC). **(B)** The moving tracks of the place navigation test and spatial probe test. **(C–E)** The swimming speed of the mice, percent of time the mice spent in the target quadrant, and the number of times the mice crossed to the platform during the spatial probe test. The data are presented as the mean ± SD of 10 mice per group. ^∗∗^*P* < 0.01.

Assessment of cognitive impairments is the typical method to evaluate the neurological injury of mice with *A. cantonensis* infection. The changes in escape latency onto a hidden platform obtained by spatial memory training trials were shown in Figures 2A,B. Compared to the control group, the escape latencies of *A. cantonensis* infected mice were significantly delayed. Treatment with TSA significantly decreased the impairment of spatial learning memory in infected mice with shorter escape latency. The results of the probe test also showed significant cognitive impairment in the *A. cantonensis*-infected mice (Figures 2B–E). The average swimming speed and the percentage of time spent in the target quadrant significantly decreased in the infected group as compared to the control. This effect was significantly reversed in infected mice that received TSA treatment. The times of crossing over the platform site of mice in the control and TSA treatment groups were higher than the *A. cantonensis* infected group, although with no significant difference among groups. Thus, cognitive impairments in *A. cantonensis* infected mice, including sense, motor, learning, and memory, were ameliorated in infected mice that received TSA treatment. Noteworthy, the extent of improvement in cognitive impairment of infected mice exhibited by the high-dose TSA group was not as great as that of the low-dose group.

### TSA Treatment Reduced the Inflammatory Factors in Mice Induced by *A. cantonensis*

**FIGURE 3:**
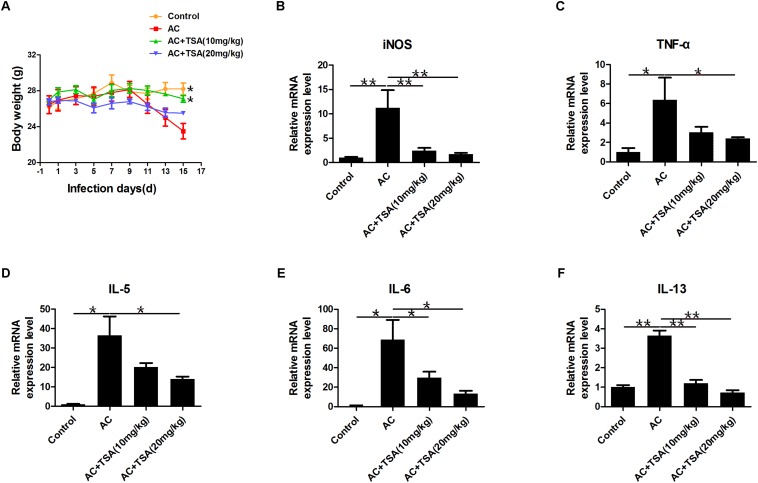
TSA treatment alleviated inflammatory responses induced by *A. cantonensis* in mice. **(A)** The changes of body weight in infected mice with TSA treatments (^∗^*P* < 0.05, control versus AC, AC + TSA (10 mg/kg) versus AC). **(B–F)** The relative mRNA expression of iNOS, TNF-α, IL-5, IL-6, and IL-13 in infected mouse brains with TSA treatments. The data are presented as the mean ± SD, *n* = 7. ^∗^*P* < 0.05, ^∗∗^*P* < 0.01.

TSA treatment alleviated the loss of body weight in infected mice from the 13th dpi compared to control (Figure 3A). The pathological responses were also significantly alleviated in infected mice that received 10 and 20 mg/kg TSA treatments. The expression of inflammatory cytokines was further observed to determine the effects of TSA on eosinophilic meningitis in infected mice. TNF-α, IL-5, IL-6, and IL-13 are typical inflammatory cytokines released in response to eosinophilic meningitis in infected mice (Kopf et al., 1996; Mishra and Rothenberg, 2003; Intapan et al., 2008; Chuang et al., 2010). As shown in Figures 3B–F, the increased levels of inducible nitric oxide synthase (iNOS), TNF-α, IL-5, IL-6, and IL-13 in infected mice were significantly decreased after 10 and 20 mg/kg TSA treatments. Thus, TSA treatment might have a protective effect on eosinophilic meningitis induced by *A. cantonensis* in mice.

### TSA Treatment Counteracts Apoptosis in Mice Infected With *A. cantonensis*

**FIGURE 4:**
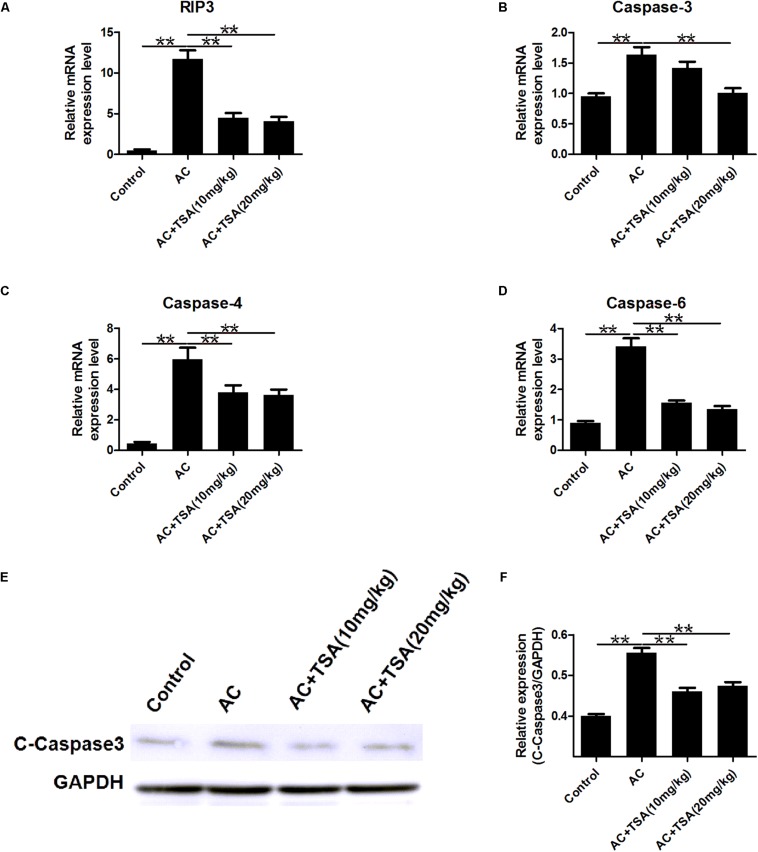
TSA treatment counteracts apoptosis induced by *A. cantonensis* in mice. **(A–D)** The relative mRNA expression of caspase-3, -4, -6, and RIP3 in mouse brains (mean ± SD, *n* = 7, ^∗∗^*P* < 0.01). **(E,F)** Western blotting revealed the expression level of cleaved caspase-3 in mouse brains. The data are presented as the mean ± SD, *n* = 3. ^∗∗^*P* < 0.01.

To further detect the impact of TSA on the apoptotic effects induced by *A. cantonensis* in the mouse brain, the mRNA levels of caspase-3, -4, -6, and receptor-interacting protein kinase 3 (RIP3) related to apoptosis and necroptosis were measured. The mRNA levels of caspase-3, -4, -6, and RIP3 in the infected mouse brains were higher than those in the control, and the higher expression levels of these molecules were reduced by treatment with TSA (Figures 4A–D). We also observed that the cleaved caspase-3 protein level was higher in infected mice than in infected mice that received TSA treatment (Figures 4E,F).

### Inhibitory Effect of TSA on HDAC With *A. cantonensis* Infection in Mice

**FIGURE 5:**
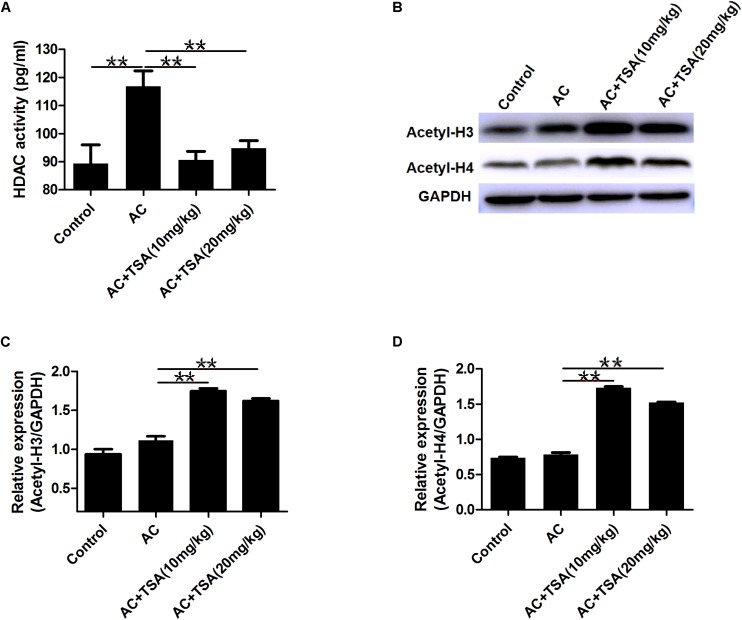
TSA treatment reduced HDAC activity and promoted histones acetylation. **(A)** TSA treatment reduced total HDAC activity in serum of infected mice (mean ± SD, *n* = 7, ^∗∗^*P* < 0.01). **(B)** Western blotting showed TSA treatment promoted the acetylation of histone (H3 and H4) in the mouse brain. **(C,D)** Relative expressions of acetyl-H3 and acetyl-H4, respectively. The data are presented as the mean ± SD, *n* = 3. ^∗∗^*P* < 0.01.

TSA treatment effectively inhibited the increased HDAC activity in serum of mice induced by *A. cantonensis* infection (Figure 5A). We also analyzed the acetylation status of histone H3 lysine 9 and histone H4 lysine 8 in brain tissues to further evaluate the inhibitory effect of TSA on HDAC in infected mice. Western blotting analyses showed that the levels of acetyl-H3 and acetyl-H4 significantly increased in infected mice that received TSA treatment (Figures 5B–D).

### TSA Suppressed NF-κB p65 Protein Nuclear Accumulation

**FIGURE 6:**
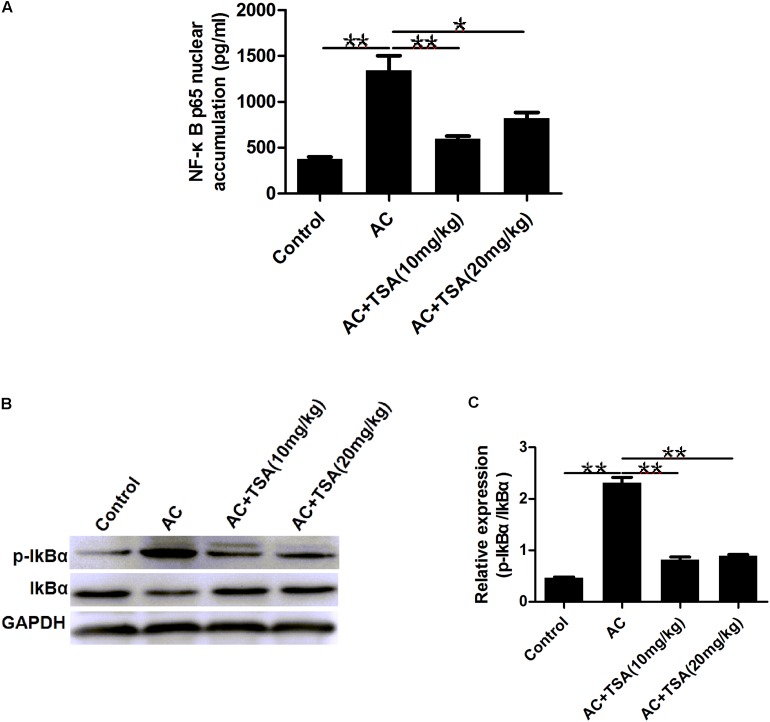
TSA suppressed the NF-κB p65 protein nuclear accumulation. **(A)** The NF-κB p65 nuclear accumulation of brain nuclear extracts was measured using the NF-κB p65 assay kit (mean ± SD, *n* = 7. ^∗^*P* < 0.05, ^∗∗^*P* < 0.01). **(B,C)** Western blotting showed the expression level of total IκB and phosphorylated IκBα in the mouse brain. The data are presented as the mean ± SD, *n* = 3. ^∗∗^*P* < 0.01.

To determine whether the anti-inflammatory effect of TSA was correlated with the NF-κB signaling pathway, we measured the accumulation of the NF-κB subunit p65 in the nucleus. Mice infected with *A. cantonensis* exhibited a high nuclear accumulation of NF-κB p65 compared to the control group, and this accumulation was partially inhibited by treatment with TSA (Figure 6A). The IκB kinase (IKK) was examined as another potential regulator of NF-κB. TSA inhibited IκB degradation in mice resulting from *A. cantonensis* infection (Figures 6B,C). In addition, the amount of phospho-IκB (p-IκB) increased in mice with *A. cantonensis* infection, and this phosphorylation was also inhibited by TSA treatment (Figures 6B,C). These results suggested that the protective effect of TSA on the progression of eosinophilic meningitis in an *A. cantonensis* infected model may be involved in inhibiting the NF-κB pathway.

### Co-expression Network of *NF-*κ*B p65* in Mice Induced by *A. cantonensis*

**FIGURE 7:**
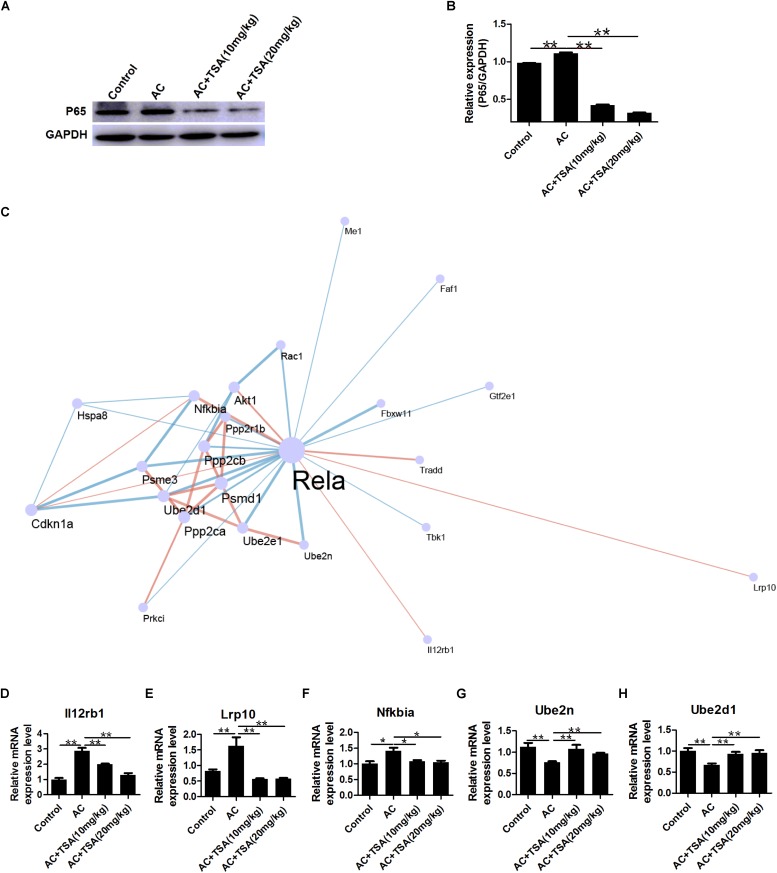
Expression profiling of genes interacting with NF-κB p65 in mice induced by *A. cantonensis*. **(A,B)** Western blotting showed the expression level of NF-κB p65 in mouse brains (mean ± SD, *n* = 3. ^∗∗^*P* < 0.01). **(C)** A sub network (center) of genes interacting with *NF-*κ*B p65* (*Rela*) was identified by integrating the String database and RNA-seq of mice with *A. cantonensis* at 2, 7, 14, and 21 dpi. Pink line: positive correlation; blue line: negative correlation. **(D–H)** Expression profile of *Lrp10, Il12rb1, Nfkbia*, *Ube2n*, and *Ube2d1* genes interacting with *NF-*κ*B p65* in mice with *A. cantonensis* infection. The data are presented as the mean ± SD, *n* = 7. ^∗^*P* < 0.05, ^∗∗^*P* < 0.01.

*NF-*κ*B p65* expression can be activated by *A. cantonensis* infection in mice, but *NF-*κ*B p65* expression significantly decreased in infected mice that received TSA treatment (Figures 7A,B). NF-κB is a typically inflammatory effector that regulates the apoptotic and inflammatory responses by controlling a fraction of cytokines (Chen and Greene, 2004; Place et al., 2005; Leus et al., 2016). Thus, analysis of cytokines co-expression with *NF-*κ*B p65* might provide some information regarding murine eosinophilic meningitis induced by *A. cantonensis*. A co-expression network of *NF-*κ*B p65* and 22 other genes was constructed for infected mice according to our previous transcriptome data (Figure 7C and Supplementary Table S2). The profile of gene expression in the co-expression network was further identified in infected mice after TSA treatment by the qPCR method. The results showed that the interleukin 12 receptor subunit beta 1 (*Il12rb1*), LDL receptor related protein 10 (*Lrp10*), and NF-κB-inhibitor alpha (*Nfkbia*) were positively correlated with *NF-*κ*B p65* expression and ubiquitin-conjugating enzyme E2 N (*Ube2n*) and ubiquitin-conjugating enzyme E2 D1 (*Ube2*d1) were negative correlation with *NF-*κ*B p65* in infected mice even after TSA treatment (Figures 7D–H and Supplementary Figure S1). We also identified the potential TFBS of NF-κB p65 on the promoter region of these genes (Supplementary Table S3). The results indicated that there is a considerably high score for NF-κB-related TFBS found in the promoters of these genes. Thus, NF-κB p65 might play the essential role in regulating the TSA effects on the eosinophilic meningitis induced by *A. cantonensis* in mice.

## Discussion

Eosinophilic meningitis in mice induced by *A. cantonensis* is characterized by infiltration of eosinophil and inflammatory cells and neuronal apoptosis (Luo et al., 2017). HDACi has long been used to treat neurodegenerative diseases (Hahnen et al., 2008). Here, we investigated the effect of TSA on mice eosinophilic meningitis induced by *A. cantonensis*. The results showed that TSA treatment alleviated the loss of body weight, relieved the meningitis, and ameliorated cognitive impairments in infected mice by increasing acetylation levels of H3 and H4. These findings were in accordance with the anti-inflammatory effects of HDACi observed in other diseases (Moreira et al., 2003; Kim et al., 2007; Shein et al., 2009).

Mice with *A. cantonensis* infection exhibited no significant changes in the acetylation levels of H3 and H4, although increased HDAC activity was observed (Figure 5). We propose that increased expression of KAT2a and HADC in infected mice (Supplementary Table S2) might maintain the balance of the acetylation levels of H3 and H4. In the current study, inhibition of HDAC activities by TSA treatment interfered with the balance by decreasing the HDAC activity and significantly increasing the acetylation levels of H3 and H4 in infected mice. These results are consistent with those of previous study of TSA-treated mice with increased acetylation levels of H3 and H4 (Avila et al., 2007).

TSA treatment ameliorates the pathological changes in infected mice with decreased expression of some cytokines, including TNF-α, iNOS, IL-5, IL-6, and IL-13. These cytokines are typical inflammatory factors that are produced in eosinophilic meningitis (Kopf et al., 1996; Mishra and Rothenberg, 2003; Intapan et al., 2008; Chuang et al., 2010). Our results indicated that TSA treatment alleviated eosinophilic meningitis of infected mice by decreasing the expression of some typical inflammatory cytokines. Previous studies also showed that inhibition of HDAC activity could alleviate eosinophilic meningitis and reduce the expression of the cytokines TNF-a, IL-1β, IL-6, IL-10, IL-12, IL-18, and iNOS (Leoni et al., 2002; Yu et al., 2002; Zhang et al., 2015).

Clinical exploration and animal studies confirmed that *A. cantonensis* invading the central nervous system caused neurological manifestations with behavioral dysfunctions (Hidelaratchi et al., 2005; Luo et al., 2017; Mengying et al., 2017). In the current study, we clearly elucidated that *A. cantonensis* infected mice exhibited dysfunctions of movement and cognition in a water maze task. TSA treatment attenuated these memory impairments induced by *A. cantonensis* in mice. It is known that memory loss and cognitive impairment are always closely correlated with neuronal apoptosis. In our study, TSA treatment counteracted apoptosis with dramatically decreased levels of cleaved caspase-3, -4, -6, and RIP3 in the brain tissue of infected mice. This result was consistent with the effects of neuroprotection by TSA treatment in some previous studies (Camelo et al., 2005; Fleiss et al., 2012).

NF-κB plays a central role in the regulation of gene expression of various cytokines, chemokines, and adhesion molecules, which are involved in inflammation, immune responses, and cell survival (Chen and Greene, 2004). NF-κB could regulate the cell apoptosis involved in the transcriptional regulation of various apoptosis-related genes (Baichwal and Baeuerle, 1997; Biswas et al., 2004). The NF-κB p65 nuclear accumulation through the phosphorylation of IκB induced subsequent degradation and the release of p50/p65 complexes (Anest et al., 2003). Studies have shown that the development of brain injury with eosinophilia induced by *A. cantonensis* infection is also associated with the IκB kinase (IKK)/NF-κB pathway (Lan et al., 2007; Chiu and Lai, 2013; Chen and Wang, 2017). It is well-known that HDAC inhibitors prevent the degradation of IκB to affect the NF-κB p65 nuclear accumulation (Place et al., 2005; Nam et al., 2010). In our study, accumulation of the NF-κB subunit p65 in the nucleus markedly decreased, and phosphorylation of IκB also decreased following TSA treatment in *A. cantonensis-*infected mice. All the results suggest that TSA, the HDAC inhibitor, might retard the progression of eosinophilic meningitis and apoptosis of infected mice through the NF-κB signaling pathway.

We further analyzed the co-expressive network of NF-κB that regulates eosinophilic meningitis induced by *A. cantonensis* infection. Twenty-two genes were recruited in the co-expressive network of NF-κB p65 in infected mice according to our previous transcriptome data. After infected mice were treated with TSA, five genes of *Lrp10, Il12rb1, Nfkbia, Ube2n*, and *Ube2d1* remained in accordance with the expression profile of NF-κB p65. Thus, these genes might be coordinated to regulate the effect of TSA on eosinophilic meningitis in infected mice.

The expression profiles of *Il12rb1*, *Lrp10*, and *Nfkbia* were similar to that of *NF-κB p65*. *Il12rb1* promotes IFN-γ immunity, mycobacterial disease resistance, and T cell differentiation (Chua et al., 1995; Robinson et al., 2010; Bustamante et al., 2014; Reeme et al., 2019). The main function of *Il12rb1* is cooperating with the proinflammatory cytokines interleukin-12 and interleukin-23 (IL12/23) to stimulate the signaling pathways. Lrp10 (murine Lrp9), a member of the low-density lipoprotein (LDL) receptor family, is expressed in various tissues and is located in endosomes and in the trans-Golgi network (TGN) (Sugiyama et al., 2000; Boucher et al., 2008; Doray et al., 2008). Knockdown of *Lrp10* led to an increase in the processing of amyloid precursor protein (APP) to Aβ peptide (Brodeur et al., 2012). Lrp10 protects APP in amyloidogenic processing, but little is known about its other functions (Brodeur et al., 2012). However, the relationship between *Il12rb1*, *Lrp10*, and *NF-κB* in inflammatory responses and neurological diseases remains unknown. It is well-documented that the protein product of *Nfkbia* mRNA is IκBa, which is the main negative regulator of NF-κB activation, while NF-κB activates Nfκbia transcription (Brown et al., 1993; Malek et al., 1998; Nelson et al., 2004; Gao et al., 2005; Ferreiro and Komives, 2010). Thus, the intra-cellular regulatory loop in the NF-κB signaling pathway is activated in regulating eosinophilic meningitis in infected mice that received TSA treatment.

As for negative relationships with expression of genes with *NF-*κ*B p65*, *Ube2n*, and *Ube2d1* genes belong to the ubiquitin-conjugating E2 enzyme (UB-E2) families in the ubiquitin-proteasome system. Ube2n and Ube2d1 transfer the ubiquitin to target proteins to regulate the ubiquitylation of proteins (Ye and Rape, 2009). Ubiquitination is the post-translational modification of proteins, and it plays a critical role in regulating protein degradation, protein trafficking, DNA repair, and signal transduction (Yamamoto et al., 2006; Ye and Rape, 2009). Ube2n forms a heterodimer with Uev1A to positively regulate the NF-κB signal-transduction pathway (Mukhopadhyay and Riezman, 2007). Although there is only limited information on the relationship of NF-κB and UB-E2, it provides us with clues to further study the regulatory functions of these genes involved in eosinophilic meningitis of infected mice.

In summary, we demonstrated that TSA treatment has beneficial effects on brain injury in mice infected with *A. cantonensis* by inhibiting the release of some inflammatory cytokines and apoptosis. The underlying mechanism of TSA on eosinophilic meningitis might be associated with the NF-κB pathway. Furthermore, co-expression analysis provided us with some potential genes that regulate eosinophilic meningitis. This study implies that TSA has a protective effect on the progression of eosinophilic meningitis induced by *A. cantonensis* infection.

## Data Availability Statement

All datasets generated for this study are included in the manuscript/Supplementary Files.

## Ethics Statement

All procedures involving animals conformed to the Chinese National Institute of Health Guide for the Care and Use of Laboratory Animals, and the protocol was approved by the Sun Yat-sen University Committee for Animal Research (No. 2016-104).

## Author Contributions

YZ, HX, WT, XZ, and YL performed the experiments. YZ and DY contributed to the data analysis. LXi and JX provided some suggestions on experiments and writing. ZW and DY designed the study. ZW, DY, and YZ wrote the manuscript. All authors revised and approved the manuscript.

## Conflict of Interest

The authors declare that the research was conducted in the absence of any commercial or financial relationships that could be construed as a potential conflict of interest.

## Footnotes

1 https://string-db.org/

2 http://jaspar.genereg.net/analysis
