# Supplementary material for: Trichostatin A, a Histone Deacetylase Inhibitor, Alleviates Eosinophilic Meningitis Induced by Angiostrongylus cantonensis Infection in Mice
Source: Front Microbiol. 2019 Oct 4;10:2280. doi: 10.3389/fmicb.2019.02280 (PMC6787401; doi:10.3389/fmicb.2019.02280)
Supplement: Supplementary file 1 [file Table_1.DOCX]

| **Supplementary Table S1. Primers Used for RT-PCR Analysis.** | | |
| --- | --- | --- |
| **Genes** | **Primer** | **sequence (5′→3′)** |
| iNOS | Forward primer | ACCTTGTTCAGCTACGCCTT |
|  | Reverse primer | CATTCCCAAATGTGCTTGTC |
| TNF-α | Forward primer | GAACTGGCAGAAGAGGCACT |
|  | Reverse primer | AGGGTCTGGGCCATAGAACT |
| IL-5 | Forward primer | TGTGAAATGCCACCTTTTGA |
|  | Reverse primer | TGTCCTCATCCTGGAAGGTC |
| IL-6 | Forward primer | CTGATGCTGGTGACAACCAC |
|  | Reverse primer | CAGAATTGCCATTGCACAAC |
| IL-13 | Forward primer | AGCATGGTATGGAGTGTGGA |
|  | Reverse primer | TTGCAATTGGAGATGTTGGT |
| β-actin | Forward primer | GGCATCCTGACCCTGAAGTA |
|  | Reverse primer | CTCTCAGCTGTGGTGGTGAA |
| Lrp10 | Forward primer | TGCACCTGCAAACTCTATGC |
|  | Reverse primer | AGAACGTAGGTTTCCCAGCA |
| Rac1 | Forward primer | CGAAAGAGATCGGTGCTGTC |
|  | Reverse primer | ACAGAGAACCGCTCGGATAG |
| Psme3 | Forward primer | AAGCGCAGGTTGGATGAATGT |
|  | Reverse primer | GCTGCTGGTTGCTTTTCAACA |
| Psmd1 | Forward primer | GACAACCTGCTGATGGCCTAT |
|  | Reverse primer | CGTATTGGTAGATCCAGGCAC |
| Tbk1 | Forward primer | CACATGACGGCGCATAAGAT |
|  | Reverse primer | AAGCGTCGTCCTTCGTAGAT |
| Tradd | Forward primer | CTATACGAGCAGGCCTTCCA |
|  | Reverse primer | GCCCAACAGATCCTCTGCTA |
| Il12rb1 | Forward primer | CGAATTGGACCTTGGGTGAC |
|  | Reverse primer | TGGATCTCTTGGGCCATGTT |
| Ube2n | Forward primer | TGAGAGCAACGCCCGTTATTT |
|  | Reverse primer | GCCATTGGGTATTCTTCTGGAA |
| Ube2d1 | Forward primer | CCCGTGGGAGATGACTTGTTC |
|  | Reverse primer | GGATAGTCTGTCGGAAAGTGGA |
| Cdkn1a | Forward primer | CGAGAACGGTGGAACTTTGAC |
|  | Reverse primer | CCAGGGCTCAGGTAGACCTT |
| Prkci | Forward primer | CCATGATGCCAATGGACCAG |
|  | Reverse primer | CCACTCTCCCTGGTGTTCAT |
| Ppp2ca | Forward primer | GAGGGAATCACGAGAGCAGA |
|  | Reverse primer | GTGACAGACCACCGTGTAGA |
| Fbxw11 | Forward primer | CAGAGGGGAACTACCAGAAAG |
|  | Reverse primer | CATGGGCTTCAGGTAAGAGTT |
| Hspa8 | Forward primer | CCAAGGTCCAAGTGGAATACAAA |
|  | Reverse primer | TCTTTCCGAGGTACGCTTCTG |
| Ube2e1 | Forward primer | ATGTCGGATGACGATTCGAGG |
|  | Reverse primer | TTGCTCATGCTGACTTTACTCTC |
| Faf1 | Forward primer | GGATTTTCAGGCATGTACTGGT |
|  | Reverse primer | TGCTGGATCAAATGTGGGTCC |
| Akt1 | Forward primer | AGAAGAGACGATGGACTTCCG |
|  | Reverse primer | TCAAACTCGTTCATGGTCACAC |
| Gtf2e1 | Forward primer | ATTCCACCAATCGGGCTTCC |
|  | Reverse primer | GCAATGCATAAATGGGCTCA |
| Nfkbia | Forward primer | TGAAGGACGAGGAGTACGAGC |
|  | Reverse primer | TGCAGGAACGAGTCTCCGT |
| Ppp2cb | Forward primer | GTCCGCTGTCCTGTTACCG |
|  | Reverse primer | GCTTTCGTGATTTCCTCGCAATA |
| Rela | Forward primer | GAGCCCATGGAGTTCCAGTA |
|  | Reverse primer | TTGCGCTTCTCTTCAATCCG |
| Ppp2r1b | Forward primer | TCGCGGTTTTAATCGACGAG |
|  | Reverse primer | CTACCCCGAGTGCTAGAGCTA |
| Me1 | Forward primer | GCCAATCGGAATGCATTTCGTC |
|  | Reverse primer | GGCCATAACAACCAAGTGAGC |
| RIP3 | Forward primer | AAGTGCAGATTGGGAACTACAACTC |
|  | Reverse primer | AGAATGTTGTGAGCTTCAGGAAGTG |
| Caspase-3 | Forward primer | AGCAGCTTTGTGTGTGTGATTCTAA |
|  | Reverse primer | AGTTTCGGCTTTCCAGTCAGAC |
| Caspase-4 | Forward primer | TGTCATCTCTTTGATATATTCCTGAAG |
|  | Reverse primer | CAAGGTTGCCCGATCAAT |
| Caspase-6 | Forward primer | AGACAAGCTGGACAACGTGACC |
|  | Reverse primer | CCAGGAGCCATTCACAGTTTCT |
