# Supplementary material for: Trichostatin A, a Histone Deacetylase Inhibitor, Alleviates Eosinophilic Meningitis Induced by Angiostrongylus cantonensis Infection in Mice
Source: Front Microbiol. 2019 Oct 4;10:2280. doi: 10.3389/fmicb.2019.02280 (PMC6787401; doi:10.3389/fmicb.2019.02280)
Supplement: Supplementary file 2 [file Table_2.DOCX]

| **Supplementary Table S2 Expression profiles of 23 genes, KAT and HDAC in mice with *A. cantonensis* infection.** | | | | | | | |
| --- | --- | --- | --- | --- | --- | --- | --- |
|  |  |  |  |  |  |  |  |
| **Gene ID** | **Gene Symbol** | **Gene Description** | **NO** | **2 dpi** | **7 dpi** | **14 dpi** | **21 dpi** |
| 19697 | Rela | v-rel reticuloendotheliosis viral oncogene homolog A (avian) | 9.57 | 11.44 | 12.48 | 10.65 | 14.07 |
| 18035 | Nfkbia | nuclear factor of kappa light polypeptide gene enhancer in B cells inhibitor, alpha | 21.81 | 21.8 | 28.96 | 28.11 | 40.74 |
| 65107 | Lrp10 | low-density lipoprotein receptor-related protein 10 | 18.48 | 20.34 | 23.55 | 21.39 | 25.8 |
| 71609 | Tradd | TNFRSF1A-associated via death domain | 3.28 | 5.14 | 7.34 | 5.78 | 7.95 |
| 16161 | Il12rb1 | interleukin 12 receptor, beta 1 | 0.63 | 0.65 | 0.75 | 0.58 | 1.05 |
| 12575 | Cdkn1a | cyclin-dependent kinase inhibitor 1A (P21) | 9.89 | 9.95 | 18.52 | 14.96 | 24.88 |
| 11651 | Akt1 | thymoma viral proto-oncogene 1 | 37.88 | 41.98 | 42.64 | 36.81 | 49.18 |
| 19353 | Rac1 | RAS-related C3 botulinum substrate 1 | 103.39 | 95.22 | 90.49 | 111.67 | 83.29 |
| 19192 | Psme3 | proteaseome (prosome, macropain) 28 subunit, 3 | 48.99 | 47.15 | 44.14 | 45.06 | 41.57 |
| 70247 | Psmd1 | proteasome (prosome, macropain) 26S subunit, non-ATPase, 1 | 41.2 | 38.38 | 27.97 | 39.15 | 30.03 |
| 56480 | Tbk1 | TANK-binding kinase 1 | 7.08 | 5.42 | 3.26 | 6.92 | 3.86 |
| 93765 | Ube2n | ubiquitin-conjugating enzyme E2N | 12.39 | 10.74 | 7.48 | 11.22 | 8.44 |
| 216080 | Ube2d1 | ubiquitin-conjugating enzyme E2D 1 | 33.76 | 33.13 | 26 | 35.24 | 21.14 |
| 22194 | Ube2e1 | ubiquitin-conjugating enzyme E2E 1 | 29.84 | 22.52 | 20.12 | 29.73 | 18.88 |
| 18759 | Prkci | protein kinase C, iota | 7.29 | 6.11 | 3.1 | 5.33 | 3.59 |
| 19052 | Ppp2ca | protein phosphatase 2 (formerly 2A), catalytic subunit, alpha isoform | 152.59 | 141.72 | 109.3 | 131.5 | 96.97 |
| 19053 | Ppp2cb | protein phosphatase 2 (formerly 2A), catalytic subunit, beta isoform | 49.03 | 45.97 | 41.97 | 49.98 | 40.68 |
| 73699 | Ppp2r1b | protein phosphatase 2 (formerly 2A), regulatory subunit A (PR 65), beta isoform | 1.85 | 1.65 | 1.52 | 1.82 | 1.56 |
| 103583 | Fbxw11 | F-box and WD-40 domain protein 11 | 7.4 | 6.28 | 6.1 | 6.44 | 5.91 |
| 17436 | Me1 | malic enzyme 1, NADP(+)-dependent, cytosolic | 9.03 | 9.11 | 7.38 | 9.73 | 6.33 |
| 15481 | Hspa8 | heat shock protein 8 | 347.15 | 314.39 | 235.23 | 257.67 | 187.39 |
| 14084 | Faf1 | Fas-associated factor 1 | 6.27 | 5.3 | 4.86 | 6.68 | 4.73 |
| 74197 | Gtf2e1 | general transcription factor II E, polypeptide 1 (alpha subunit) | 4.22 | 3.69 | 3.08 | 4.27 | 3.12 |
| 14534 | Kat2a | K(lysine) acetyltransferase 2A | 11.47 | 11.69 | 9.59 | 8.88 | 11.06 |
| 15182 | Hdac2 | histone deacetylase 2 | 18.92 | 20.05 | 8.86 | 19.36 | 13.40 |
| 15183 | Hdac3 | histone deacetylase 3 | 35.54 | 31.18 | 31.68 | 34.11 | 39.75 |
| 208727 | Hdac4 | histone deacetylase 4 | 3.19 | 4.79 | 2.92 | 2.11 | 1.92 |
| 15184 | Hdac5 | histone deacetylase 5 | 43.27 | 43.53 | 34.08 | 24.77 | 29.88 |
| 15185 | Hdac6 | histone deacetylase 6 | 5.24 | 5.86 | 5.53 | 4.37 | 5.06 |
| 70315 | Hdac8 | histone deacetylase 8 | 9.44 | 10.25 | 10.75 | 10.38 | 7.26 |
| 232232 | Hdac11 | histone deacetylase 11 | 115.89 | 122.40 | 110.64 | 91.59 | 121.09 |
