# Supplementary material for: Trichostatin A, a Histone Deacetylase Inhibitor, Alleviates Eosinophilic Meningitis Induced by Angiostrongylus cantonensis Infection in Mice
Source: Front Microbiol. 2019 Oct 4;10:2280. doi: 10.3389/fmicb.2019.02280 (PMC6787401; doi:10.3389/fmicb.2019.02280)
Supplement: Supplementary file 3 [file Table_3.DOCX]

**Supplementary Table S3** NF-κB TFBS found in promoter region of the five genes of *Lrp10, Il12rb1, Nfkbia, Ube2n* and *Ube2d1*.

| Gene name | Association score | Predicted sequence |
| --- | --- | --- |
| Il12rb1 | 8.2035 | GGGACATTCA |
|  | 6.85251 | TGGAATTCAC |
| Ube2n | 9.79626 | GGGACTTCCA |
|  | 7.85824 | GGGACTGTCT |
|  | 7.21724 | GCGACGTTCC |
|  | 7.03809 | GGGGATTACA |
| Ube2d1 | 8.92817 | GGGGCTTCCA |
| Nfkbia | 9.54353 | GGGAAAATCC |
|  | 9.37611 | GGGACACCCC |
