# Supplementary material for: Trichostatin A, a Histone Deacetylase Inhibitor, Alleviates Eosinophilic Meningitis Induced by Angiostrongylus cantonensis Infection in Mice
Source: Front Microbiol. 2019 Oct 4;10:2280. doi: 10.3389/fmicb.2019.02280 (PMC6787401; doi:10.3389/fmicb.2019.02280)
Supplement: Supplementary file 4 [file Data_Sheet_1.PDF]

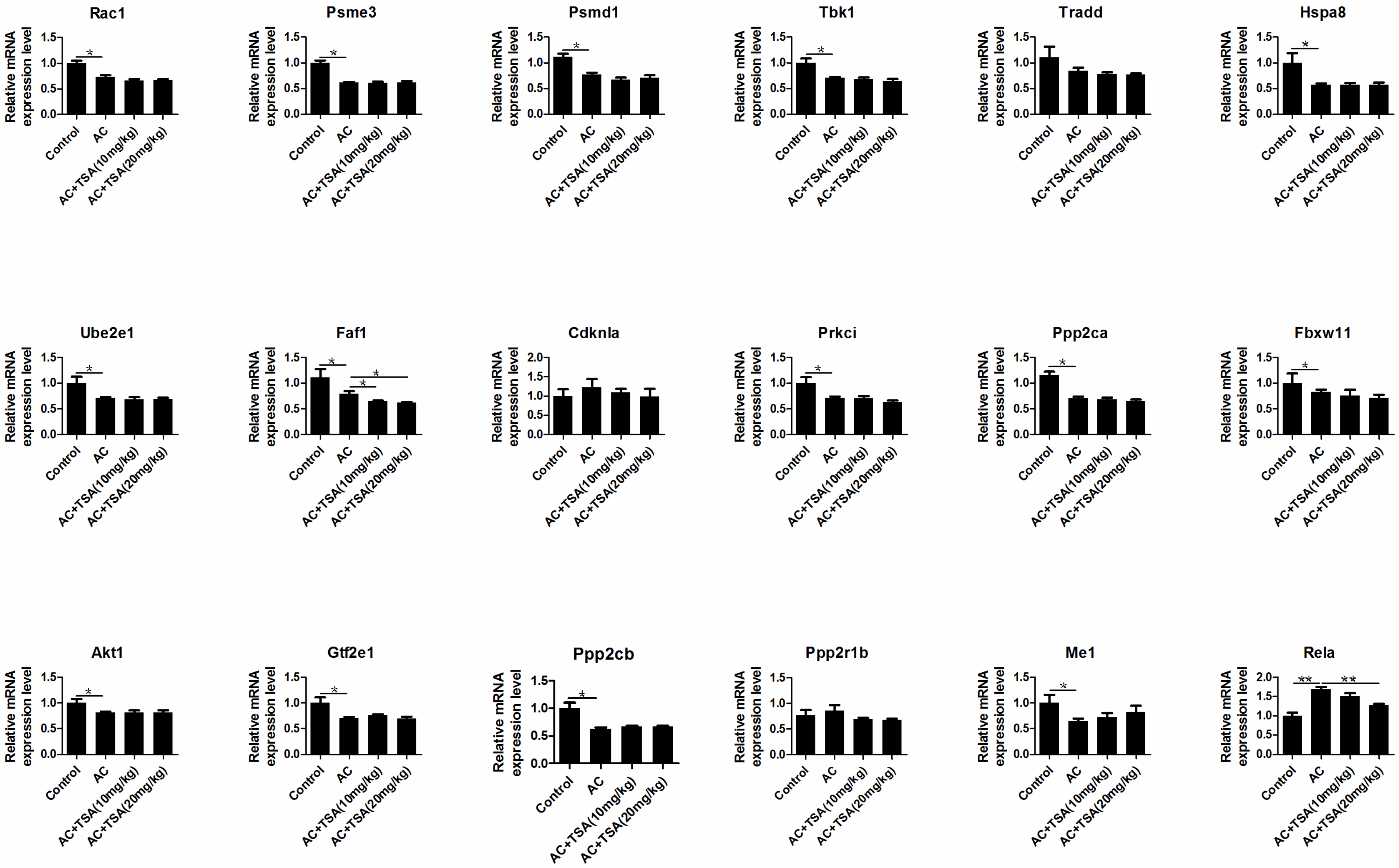

Supplementary Figure S1: Expression profile of the other 17 genes interacting with *NF-κB p65* with *A. cantonensis* infection in mice (**P*<0.05, ***P*<0.01).
